# *Limnospira* (Cyanobacteria) chemical fingerprint reveals local molecular adaptation

**DOI:** 10.1128/spectrum.01901-24

**Published:** 2025-01-08

**Authors:** Théotime Roussel, Cédric Hubas, Sébastien Halary, Mathias Chynel, Charlotte Duval, Jean-Paul Cadoret, Tarik Meziane, Léa Vernès, Claude Yéprémian, Cécile Bernard, Benjamin Marie

**Affiliations:** 1 UMR7245 MCAM MNHN-CNRS, Muséum National d’Histoire Naturelle, Paris, France; 2 Algama, Paris, France; 3 UMR 7208 BOREA MNHN-CNRS-SU-IRD, Muséum d’Histoire Naturelle, Paris, France; University of Porto, Porto, Portugal

**Keywords:** cyanobacteria, metabolomics, genomics, adaptation, micro-diversity

## Abstract

*Limnospira* can colonize a wide variety of environments (e.g., freshwater, brackish, alkaline, or alkaline-saline water) and develop dominant and even permanent blooms that overshadow and limit the diversity of adjacent phototrophs, especially in alkaline and saline environments. Previous phylogenomic analysis of *Limnospira* allowed us to distinguish two major phylogenetic clades (I and II) but failed to clearly segregate strains according to their respective habitats in terms of salinity or biogeography. In the present work, we attempted to determine whether *Limnospira* displays metabolic signatures specific to its different habitats, particularly brackish and alkaline-saline ecosystems. The impact of accessory gene repertoires on respective chemical adaptations was also determined. In complement of our previous phylogenomic investigation of *Limnospira* (Roussel et al., 2023), we develop a specific analysis of the metabolomic diversity of 93 strains of *Limnospira*, grown under standardized lab culture conditions. Overall, this original work showed distinct chemical fingerprints that were correlated with the respective biogeographic origins of the strains. The molecules that most distinguished the different *Limnospira* geographic groups were sugars, lipids, peptides, photosynthetic pigments, and antioxidants. Interestingly, these molecular enrichments might represent consequent adaptations to conditions of salinity, light, and oxidative stress in their respective sampling environments. Although the genes specifically involved in the production of these components remain unknown, we hypothesized that within extreme environments, such as those colonized by *Limnospira*, a large set of flexible genes could support the production of peculiar metabolite sets providing remarkable adaptations to specific local environmental conditions.

**IMPORTANCE:**

*Limnospira* are ubiquitous cyanobacteria with remarkable adaptive strategies allowing them to colonize and dominate a wide range of alkaline-saline environments worldwide. Phylogenomic analysis of *Limnospira* revealed two distinct major phylogenetic clades but failed to clearly segregate strains according to their habitats in terms of salinity or biogeography. We hypothesized that the genes found within this variable portion of the genome of these clades could be involved in the adaptation of *Limnospira* to local environmental conditions. In the present paper, we attempted to determine whether *Limnospira* displayed metabolic signatures specific to its different habitats. We also sought to understand the impact of the accessory gene repertoire on respective chemical adaptations.

## INTRODUCTION

Certain phytoplankton can sustainably colonize a specific environmental niche. This capability appears to be balanced by their specific ability to perform efficient primary production, to avoid being overshadowed by competitors, and to deal with physico-chemical environmental constraints. This adaptive power is strongly dependent on the energy they are capable of expending relative to the genomic or physiological cost (1).

Cyanobacteria are photoautotrophic microorganisms that have colonized a wide range of environments around the world (2, 3). The large diversity of cyanobacteria species is accompanied by a great diversity of primary and secondary metabolites, many of which have specialized biological activities in key cellular and ecological functions (4, 5). The genus *Limnospira* (anc. *Arthrospira*), better known under its commercial name “spirulina,” includes some of the most extensively researched cyanobacterial taxa studied so far (*>*300 publications per year since 2020 on PubMed), especially because of its industrial potential as an agri-food, in pharmaceuticals, and in cosmetics (6, 7). Cultured *Limnospira* possess a remarkably high growth rate of more than one division per day under optimal culture conditions (8, 9) and are very rich in protein (up to 70% of dry weight) (10), unsaturated fatty acids (11), vitamins (at least 9) (12, 13), and various antioxidant compounds (such as polyphenols, carotenoids, ergothioneine, and biopterins) (12, 14). In this study, the physiological capacity of specific *Limnospira* strains has been examined in terms of the effect of modifying culture conditions on biomass and production of desirable compounds (8, 15–18); only a few studies have focused on the global ecological preferences of this taxon (19).

In a recent study based on a polyphasic approach combined with comparative genomics, we showed that the *Limnospira* genus is monospecific, represented by one single species, *Limnospira platensis*, which is cosmopolitan and comprises all previously named members of *Arthrospira platensis*, *Arthrospira maxima*, *Arthrospira jenneri*, *Arthrospira indica*, *Arthrospira fusiformis*, … (20). Yet, *Limnospira platensis* seems to develop a singular ecological strategy, as this species is able to colonize a wide variety of environments (e.g., freshwater, brackish, alkaline, or alkaline-saline water) (19, 20) in which it dominates the phytoplanktonic community by developing permanent blooms that limit overshadowed adjacent phototroph diversity (19, 21).

Phylogenomic analysis of *L. platensis* allowed us to distinguish two major phylogenetic clades (I and II) but failed to clearly segregate strains according to their habitats in terms of salinity or biogeography (20). Such discrepancies have already been observed in other taxa, such as for the widespread extremophilic bacteria, *Salinibacter ruber* (22). However, in this case, analysis of non-targeted metabolites succeeded in segregating the strains and highlighting biogeographical metabolic signatures related to local adaptations. Recently, the same approach has suggested the existence of distinct chemical signatures among different ecotypes of the oceanic picocyanobacterium, *Prochlorococcus marinus*, each being adapted to a specific niche characterized in terms of light intensity and temperature (23).

Concerning the species, *L. platensis*, the flexible part of the genome represents up to 55% of the sequence of each strain and differs from one strain to another. The region has a large variety of genes, some of which have regulatory functions while others are defensive (20). Many present unknown functions that could potentially be related to specific physiological capabilities of the strains of *L. platensis* for ecological adaptation. These genes could carry traits that allow the species to adjust to specific local conditions and support the maintenance of continuing blooms. Ultimately, this local adaptation might give *L. platensis* an advantage allowing it to out-compete other phytoplankton within these environments.

In the present paper, we attempted to determine whether *L. platensis* displayed metabolic signatures specific to its varied habitats, particularly brackish or alkaline-saline ecosystems, and to analyze the impact of accessory gene repertoires on the respective chemical adaptations. To address this aim, we investigated a large panel of strains from different environments by culturing them under identical conditions. We used two types of extraction and the appropriate chemical analyses, to characterize a large group of diverse metabolites potentially involved in various adaptive traits. First, we applied a targeted approach, focusing on photosynthetic pigments and fatty acids known to be involved in adaptations to various environmental conditions including light, salinity, and pH. Then, we used an untargeted approach in parallel to obtain an overall chemical fingerprint of each strain and examine them for other potential differences.

## MATERIALS AND METHODS

### Strains and culture conditions

Ninety-three strains of *L. platensis* were studied. Eighty-nine strains were obtained from the Paris Museum Collection (PMC) (24): 72 from two Camargue areas, a humid region located in the Rhône delta (south of France) mostly consisting of marshland and brine lagoons, 13 from Lake Dziani Dzaha, an alkaline and thalassohaline lake located on the island of Petite-Terre of Mayotte, 3 from Lake Natron, an alkaline-saline lake located between Tanzania and Kenya, and 1 strain from a historical collection with no environmental data available. Three additional strains were provided from other collections: *L. platensis* PCC 8005 and *L. platensis* PCC 7345 (Pasteur Culture Collection of Cyanobacteria, France) from unknown locations and *L. platensis* SAG 85.79 (Culture Collection of Algae at Göttingen University, Germany) from Lake Natron. One strain, Paracas R14 from a freshwater lake in Peru, was provided by Spirulina Solutions (https://spirulinasolutions.fr/). All information about strains, sampling site properties, year of isolation, genome accession numbers, and other genomic information is given in Table S1. These 93 strains were maintained as monoclonal but non-axenic cultures, growing in 30 mL of improved Spirulina medium (24) at 24°C with a photon flux density of 30 µmol photon∙m^−2^∙s^−1^ under a 16:8 h light:dark photoperiod. When determining the metabolite production of the various strains, careful attention was paid to maintaining cultures in the exponential phase to keep the level of heterotrophic bacteria as low as possible. Although *Limnospira* produces a reduced amount of exopolysaccharides that can potentially be colonized by such heterotrophic bacteria, we performed direct observation of cultures by photonic microscope to ensure it remains negligible compared with the cyanobacterial population—in terms of biovolume and subsequent biomass and metabolite contents—and would not influence our conclusions from the observations on *Limnospira* strain distinctions. Each month, a volume equivalent to 10% of each culture was used to inoculate new cultures. The remaining 90% of cells was washed at room temperature three times with pure water by centrifugation for 10 min at 3,220 *g*. The supernatants were discarded, and the pellets were stored at −80°C for at least 3 h, and then, lyophilization was performed (−47°C, 0.03 mBar for 16 h) (Labconco, Kansas City, MO, USA). Six successive cycles of culture-biomass sampling were made and pooled. The six freeze-dried biomass samples of each strain were mixed together and homogenized prior to the chemical analyses for pigments, fatty acids, and untargeted metabolites.

### Determination of pigments

Hydrophilic pigments of the 93 strains were analyzed by spectrophotometry according to Yéprémian et al. (25, 26). Ten milligrams of freeze-dried biomass was incubated with 10 mL of a saline buffer solution (NaCl 0.15 mol∙L^–1^, KCl 27 mmol∙L^–1^, Na_2_HPO_4_ 80 mmol∙L^–1^, KH_2_PO_4_ 20 mmol∙L^–1^, and Na_2_-EDTA 10 mmol∙L^–1^; pH 7.5) for 16 h, at 4°C in the dark. Samples were then centrifuged (10 min, 3,220 *g*), and 1 mL of the supernatant was analyzed with a spectrophotometer (Cary-60, Agilent) at 565, 620, and 650 nm, corrected by subtracting the value recorded at 750 nm (<0.01 absorbance unit). The values were reported as concentration (mg∙g^−1^) of C-phycocyanin and allophycocyanin (absence of phycoerythrin) and were obtained using formulas from de Marsac and Houmard (27).

Lipophilic pigments from the 93 strains were analyzed by high-performance liquid chromatography (HPLC)(28). The complete procedure is detailed in the supplementary Materials and Methods. The relative abundance of each pigment (%) was calculated from its respective concentration in the sample (mg∙g^−1^ dry weight [dw] for chlorophyll *a* derivatives and μg∙g^−1^ dw for other pigments).

### Fatty acids

Ten milligrams of freeze-dried biomass were used for the extraction of fatty acids from 91 strains (all 93 except *L. platensis* PMC 1041.18 and PMC 1044.18). Prior to extraction, an internal standard (tricosanoic acid, 23:0) was added to each sample. Lipids were extracted according to the protocol of Bligh and Dyer (29) as modified by Meziane et al. (30). The complete procedure is detailed in the supplementary Materials and Methods. Fatty acids were identified by mass spectrometry (Agilent 5977B GC/MSD) by comparison with retention times of commercial fatty acid standards (Supelco 37). We then reported the values as % of total fatty acids and concentration (mg∙g^−1^ dw).

### Mass spectrometry

The intracellular metabolite contents of 88 strains (all 93 except *L. platensis* PMC 1287.21, PMC 1288.21, PMC 1298.21, PMC 1300.21, and PMC 1302.21) were analyzed as previously described (31). The complete procedure is detailed in the supplementary Materials and Methods. Intracellular metabolite contents were analyzed using an electrospray ionization hybrid quadrupole time-of-flight high-resolution mass spectrometer (Compact, Brucker, Bremen, Germany) in the range of 50 to 1,500 *m/z*. Metabolite annotations were made from MS1 data using CyanoMetDB V1.0 (32) and from MS2 data by generating a molecular network for the comparison of fragmentation profiles using the MetGem software (version 1.3.6) with GNPS algorithm (Fig. S1).

### Statistical analyses

The MetaboAnalyst 5.0 platform (www.metaboanalyst.ca) was used to perform data matrix normalization according to *Pareto* and mean centered and divided by the standard deviation for untargeted metabolite and for fatty acid and pigment matrices, respectively.

All three matrices (annotated ions, fatty acids, and pigments) were combined in a single matrix known as the “total metabolite matrix.” The weight of each group of variables was adjusted to prevent the analysis from being influenced by the largest group. The weights were identical for the variables of the same group.

A clade based on the presence or absence of coding gene sequences was obtained with a dendrogram (according to Jaccard’s similarity/dissimilarity index, Ward reconstruction, and Silhouette analysis to find the optimal number of clusters) using R software (version 1.4.1103). Hierarchical clustering on principal components (HCPC) using Euclidian distances was performed and principal component analysis (PCA) was done to find different groups of the strain using R software (version 1.4.1103). An analysis of similarities (ANOSIM) was performed on the total metabolite matrix to test differences between factors. Multiple correspondence analysis was performed to identify correlations between tested factors using R software (version 1.4.1103). For each tested factor, multiple factor analyses (MFA) and between-class analyses (BCA) were performed simultaneously (BC-MFA code is available at https://github.com/Hubas-prog/BC-MFA) on the combined matrix, as described by Michelet et al. (33). The percentage of total inertia explained by the instrumental variable was systematically calculated. A threshold based on the square cosine of the coordinates of the variables was applied for highlighting the most discriminating variables with a squared cosine greater than 0.4 (cos^2^ > 0.4). A Monte Carlo test was systematically performed to determine the significance of the BCA ordination, using R software (version 1.4.1103). For each factor, PCA and heatmap analyses were performed on each of the three matrices using the MetaboAnalyst 5.0 platform.

Differences between each class of factor have been searched for each of the 215 molecules with Kruskal-Wallis tests followed by pairwise Wilcoxon tests. For comparison of the means of total fatty acids, normality was present (Shapiro-Wilk test, *P* > 0.05) and homogeneity of the variances was not present (Bartlett test, *P* < 0.05); thus, a Welch test was performed. All tests were run using R software (version 1.4.1103).

## RESULTS

### Pigment composition

**Fig 1:**
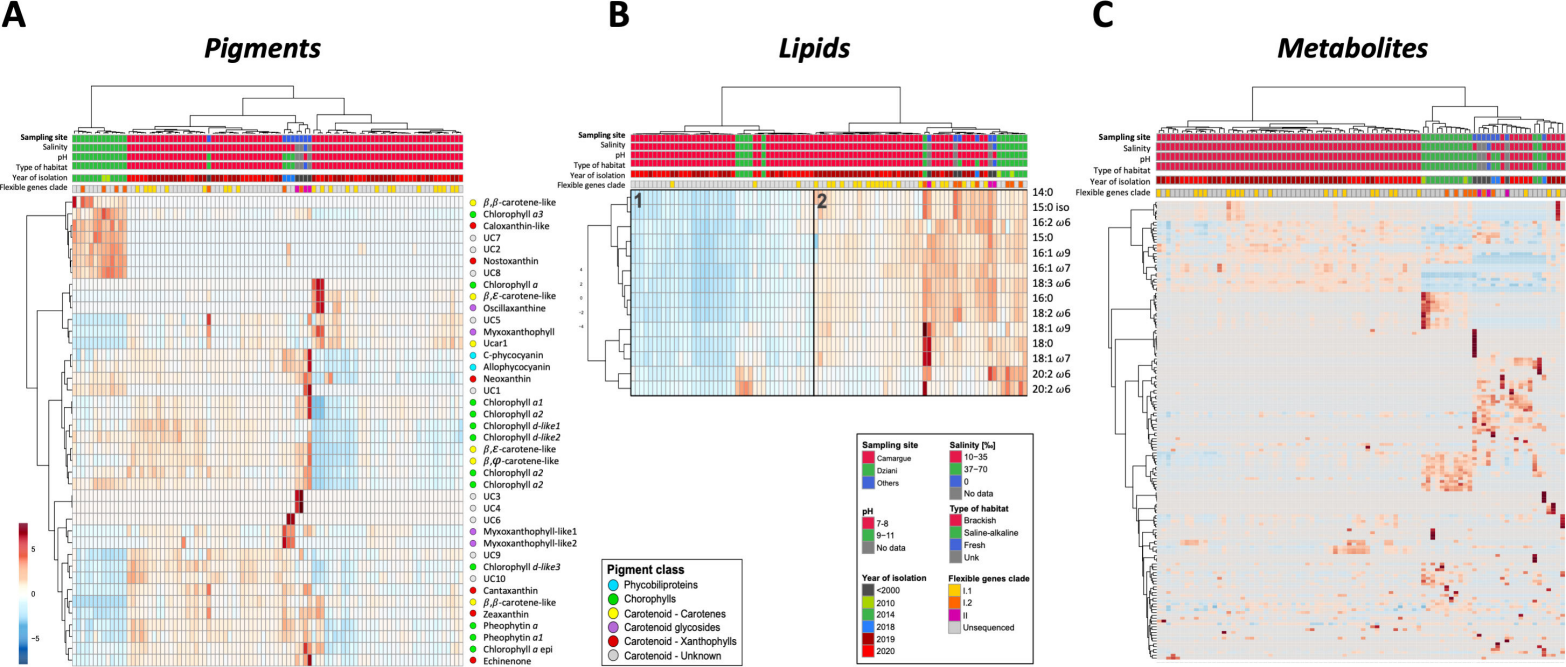
Heatmap analyses of pigment, lipid, and metabolite matrices. Heatmap analysis of the 40 pigment concentrations of the 93 analyzed *L. platensis* strains (normalization according to each feature) (**A**). Heatmap analysis of the normalized 14 fatty acid concentrations of the 91 analyzed *L. platensis* strains (**B**). Total fatty acid concentration (mg∙g^−1^ dw) discriminates between two groups of lower (1) and higher (2) lipid contents (Welch test, *P* < 0.001). (**C**) Heatmap analysis of the 161 HPLC-MS/MS annotated ions of the 88 analyzed *L. platensis* strains, expressed in normalized relative abundances. The blue-red scale indicates the semi-quantification value of each pigment, fatty acid, and metabolite normalized according to Pareto’s formula.

Forty photosynthetic pigments from the 93 analyzed *L. platensis* strains were detected and quantified by HPLC-DAD: two phycobiliproteins (C-phycocyanin and allophycocyanin), 12 chlorophylls or derivatives (chlorophylls *a*, *d*, and pheophytins), and 26 carotenoids (5 carotenes, 4 carotenoid glycosides, 6 xanthophylls, 1 unknown carotene, and 10 unknown carotenoids) (Fig. 1A; Table S2). A mean of 27.9 ± 1.8 pigments was detected per strain, ranging from 20 to 32 for strain *L. platensis* PMC 1223.20, PMC 1278.20, and PMC 1279.20 and *L. platensis* PMC 1041.18, respectively (Table S2). Twelve pigments were detected in all strains: C-phycocyanin, allophycocyanin, chlorophyll *a*, chlorophyll *a* allomer, chlorophyll *a* epimer, two pheophytins *a*, β,φ-carotene, β,ε-carotene-like, zeaxanthin, echinenone, echinenone/canthaxanthin-like, and two unknown carotenoids (UC10 and UC9) (Table S3). The most abundant pigments were C-phycocyanin (64.5% ± 3.1% of total pigment composition) and allophycocyanin (25% ± 1.6% of total pigment composition). The chlorophyll *a* allomer was the most abundant form of chlorophyll *a* in 90 out of 93 strains, representing 6.6% ± 2.3% of the total pigment composition. The three other strains (*L. platensis* PMC 1223.20, PMC 1278.20, and PMC 1279.20) had the usual form of chlorophyll *a* as the main chlorophyll pigment, representing 13.6% ± 2.3% of the total pigment composition (Table S2). Carotenoids represented 1.19% ± 0.6% of the total pigment composition. The most abundant carotenoids were zeaxanthin (34.2% ± 5.5% of the total carotenoid composition), myxoxanthophyll (14.9% ± 5.6% of total carotenoid composition), β,β-carotene (9.9% ± 1.3% of total carotenoid composition), and UC9 (425, 448, 470 nm) (8.7% ± 3.6% of total carotenoid composition) (Table S2).

### Fatty acid composition

Fourteen fatty acids were detected and quantified by GC-FID and GC-MS in the 91 analyzed *L. platensis* strains. A mean of 52.32 ± 23.77 mg fatty acids∙g^−1^ dw was quantified, ranging from 13.78 mg∙g^−1^ for *L. platensis* PMC 1259.20 to 110.59 mg∙g^−1^ for *L. platensis* PMC 894.15, respectively (Table S3). Among the 14 fatty acids detected, three were saturated (14:0, 16:0, and 18:0), two were branched (15:0iso and 15:0anteiso), four were mono-unsaturated (16:1ω7, 16:1ω9, 18:1ω7, and 18:1ω9), and five were poly-unsaturated fatty acids (16:2ω6, 18:2ω6, 18:3ω6, 20:2ω6, and 20:3ω6). Five fatty acids represented >90% of the total fatty acids: 16:0 (38.6% ± 1.8%), 18:3ω6 (24.6% ± 2.1%), 18:2ω6 (17.1% ± 1.5%), 16:1ω9 (6.2% ± 1.1%), and 16:1ω7 (5.6% ± 0.8%) (Table S3). The relative quantities of these molecules within the different strains allowed us to categorize them into groups of low and high fatty acid-containing strains according to a heatmap with hierarchical clustering and no obvious relation to the respective metadata of the strains (Fig. 1B).

### LC-MS determination of metabolite composition

A total of 2851 ions were detected by high-pressure liquid chromatography-mass spectrometry (HPLC-MS) from the 88 analyzed *L. platensis* strains. A mean of 1,311.7 ± 175.1 ions was detected, ranging from 1,023 for strain *L. platensis* PMC 1044.18 to 1,686 for *L. platensis* SAG 85.79 (Table S4). A total of 1,013 ions (35.5%) exhibited an intensity greater than the threshold of 5,000 counts in single MS and were analyzed by HPLC-MS/MS. Among them, 221 ions (15.9% fragmented ions and 5.6% total ions) were clustered and annotated with MetGem and GNPS into phospholipids, saccharides, peptides, etc. and 25 were annotated at the molecular levels into ergothioneine, glutathione, biopterin, and nicotinamide adenine dinucleotide (Fig. 1C; Table S4). Others were annotated according to the MS/MS fragmentation pattern similarity revealed by molecular networking (Fig. S1). Analyses of the 2,851 total ions and the 221 annotated ions showed similar results (*data not shown*); therefore, we focused only on the 221 annotated ions (Fig. 1C).

### Grouping of strains

**Fig 2:**
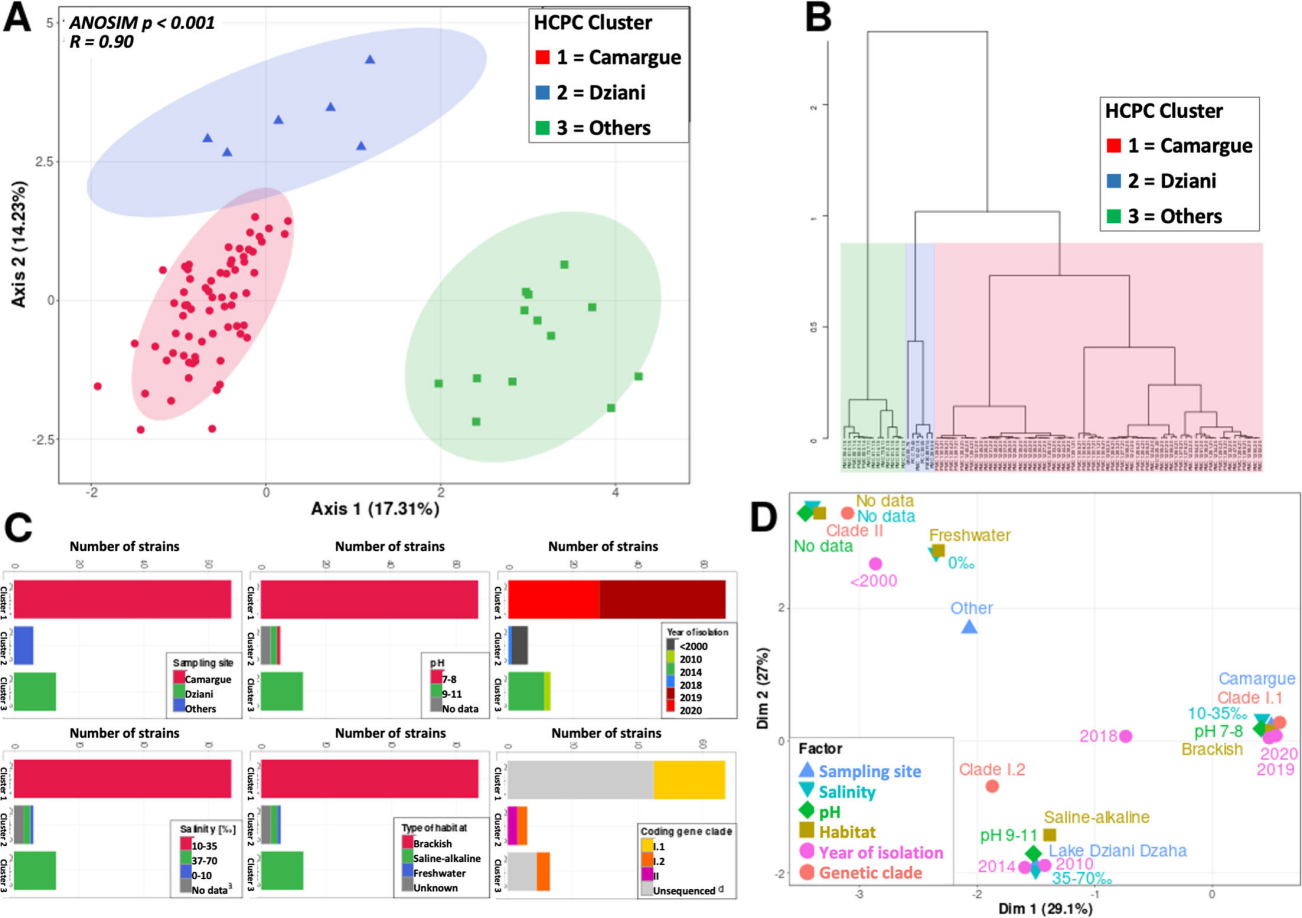
PCA (**A**). Representation of the total matrix metabolite composition of the strains for the groups given by HCPC (**B**). Characterization of each group regarding the tested factors (**C**) and the multiple correspondence analysis (**D**).

Based on the data sets containing fatty acids, pigments, and annotated ions, a complete matrix (total metabolite matrix) was assembled for performing global metabolite analyses (see Materials and Methods). A MFA combined with HCPC was performed on the total metabolite matrix. After accounting for the effect of the sampling site factor, three distinct groups of strains were identified (Fig. 2A and B, respectively). Remarkably, the three groups that were statistically different (Fig. 2A, ANOSIM *P* < 0.001) were similarly distinguishable in terms of fatty acids, pigments, and annotated ions, when considered separately (Fig. S2A through C), and overall were matched with the sampling location factor (Fig. 2C). The strains isolated from different sampling sites were clearly clustered into different groups: the Camargue region in group 1, Lake Dziani Dzaha in group 3, and another sampling site in group 2 (Fig. 2B). These analyses showed that strains coming from Lake Natron in Peru and from the three unknown locations could be arranged together into a single site group (Fig. 2A and B) named “Others.” According to the multiple correspondence analysis (Fig. 2D), the correlation between these three groups (1 = Camargue, 3 = Dziani, and 2 = others) and the different environmental factors were investigated. Group 1 tolerated a pH in the range of 7–8, salinity of 10‰–35‰, a brackish habitat, and isolation from 2019 to 2020, while Group 3 preferred a pH of 9–11, salinity of 35‰–70‰, an alkaline-saline habitat, and isolation in 2010 and 2014. Group 2 was associated with “other” pH, salinity, habitat, and year of isolation prior to 2000 (Fig. 2C and D). The three sampling site groups highlighted by the HCPC were used for further analysis of their respective metabolite signatures.

### Sampling sites

**Fig 3:**
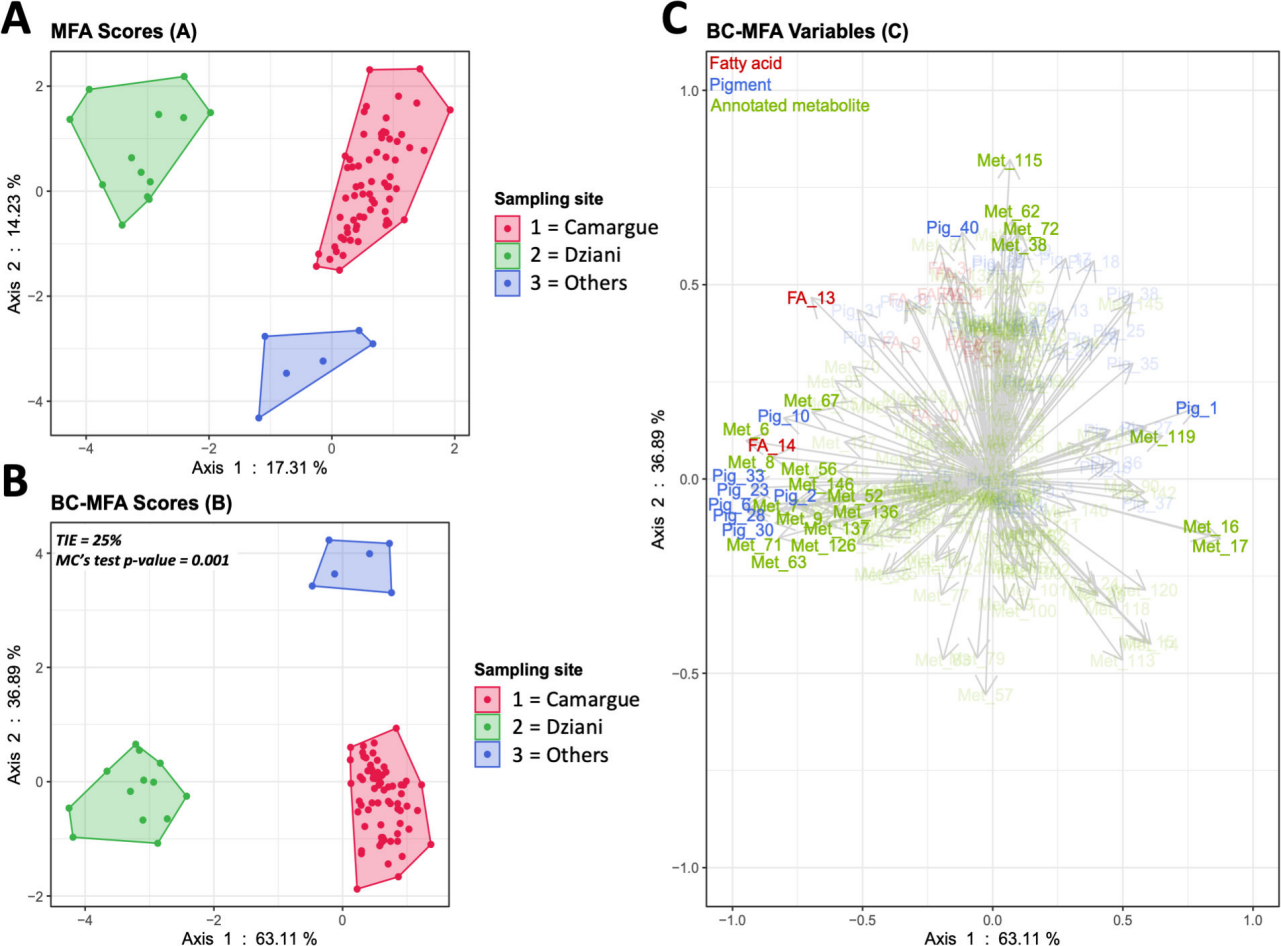
MFA (**A**), BCA with a cos^2^ threshold of 0.4 (**B**), and cos^2^ plot (**C**), based on the chemical composition of the strains. The tested factor is the sampling site.

**Fig 4:**
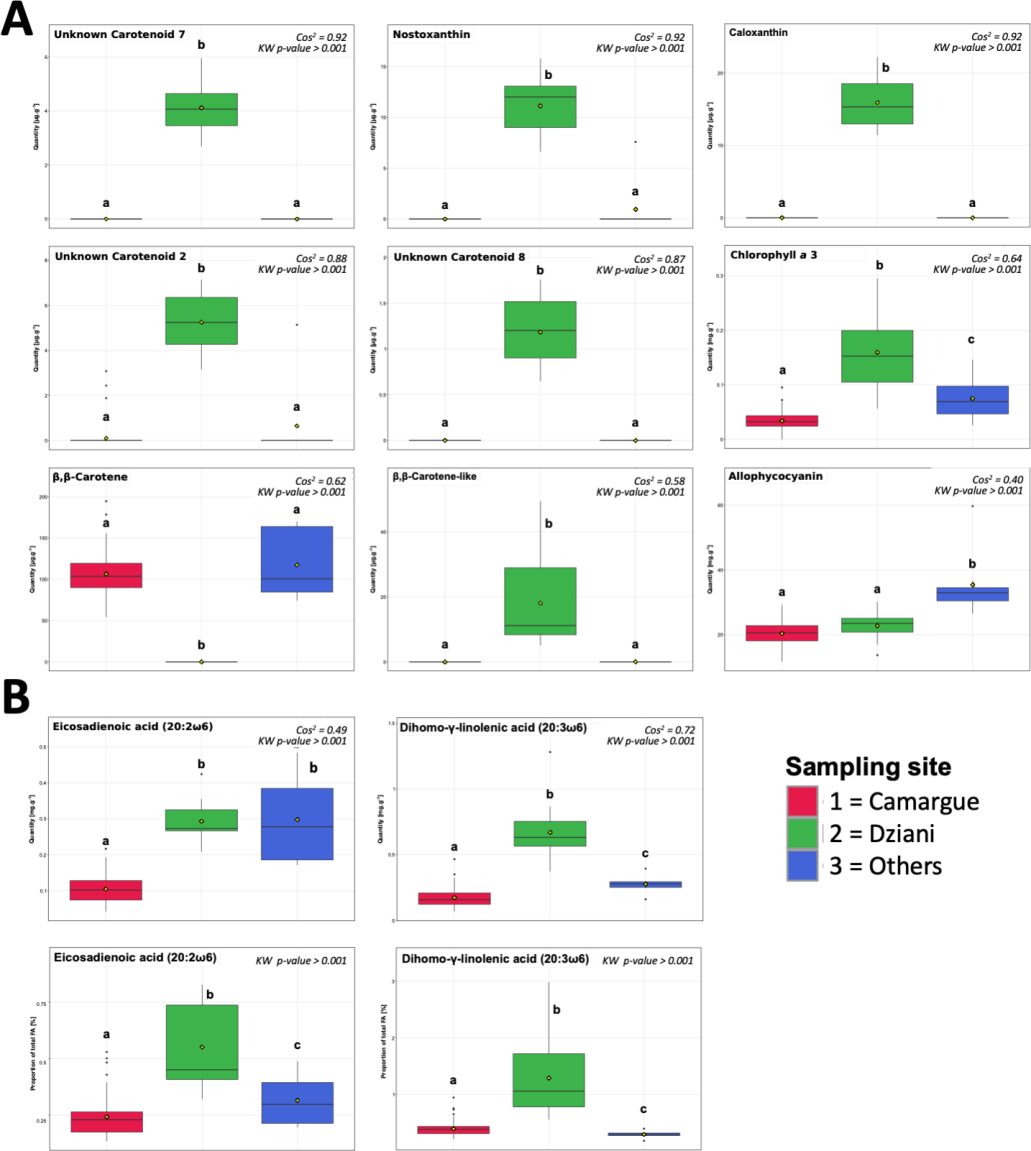
Most discriminated fatty acids and pigments (cos^2^ > 0.4) were compared between sampling sites by Kruskal-Wallis test and pairwise Wilcoxon tests. Pigment concentrations are given in mg∙g^−1^ dw for phycobiliproteins and chlorophylls *a* and µg∙g^−1^ dw for other pigments (**A**). Fatty acid concentrations are given in mg∙g^−1^ dw, and proportions in % of total fatty acids (**B**).

MFA and BCA analyses were performed on the three groups that were previously separated by HCPC and clearly corresponded to different sampling sites (Fig. 3A and B). The primary axis of the BCA analysis accounted for 63.1% of the variability, distinguishing the *L. platensis* samples taken at Dziani (group 3) from the others, mainly based on their chemical composition (Fig. 3B). The second axis was responsible for 37% of the total inertia and separated HCPC group 2 from all other strains (Fig. 3B). Thirty-one of the most discriminated variants presenting a cos^2^ > 0.4 were observed among fatty acids, pigments, and annotated ions (Fig. 3C): 2 fatty acids, 9 pigments, and 20 annotated ions (26 related to axis 1 and 5 to axis 2). In total, 126 molecules showed significant differences between at least two groups (Kruskal-Wallis test, *P* < 0.001). The two fatty acids were the polyunsaturated fatty acids (PUFAs) with the longest carbon chains, 20:2ω6 and 20:3ω6 that predominated in the strains from Lake Dziani Dzaha in terms of concentration and proportion of total fatty acids (Fig. 4, Kruskal-Wallis test, *P* < 0.001). Based on fatty acids only, two groups of 42 and 49 strains unrelated to any of the environmental factors were discriminated by hierarchal clustering, (Fig. S2B). They significantly differed by the total fatty acid biomass (30 ± 8 mg∙g^−1^ and 71 ± 15 mg∙g^−1^, respectively).

**Fig 5:**
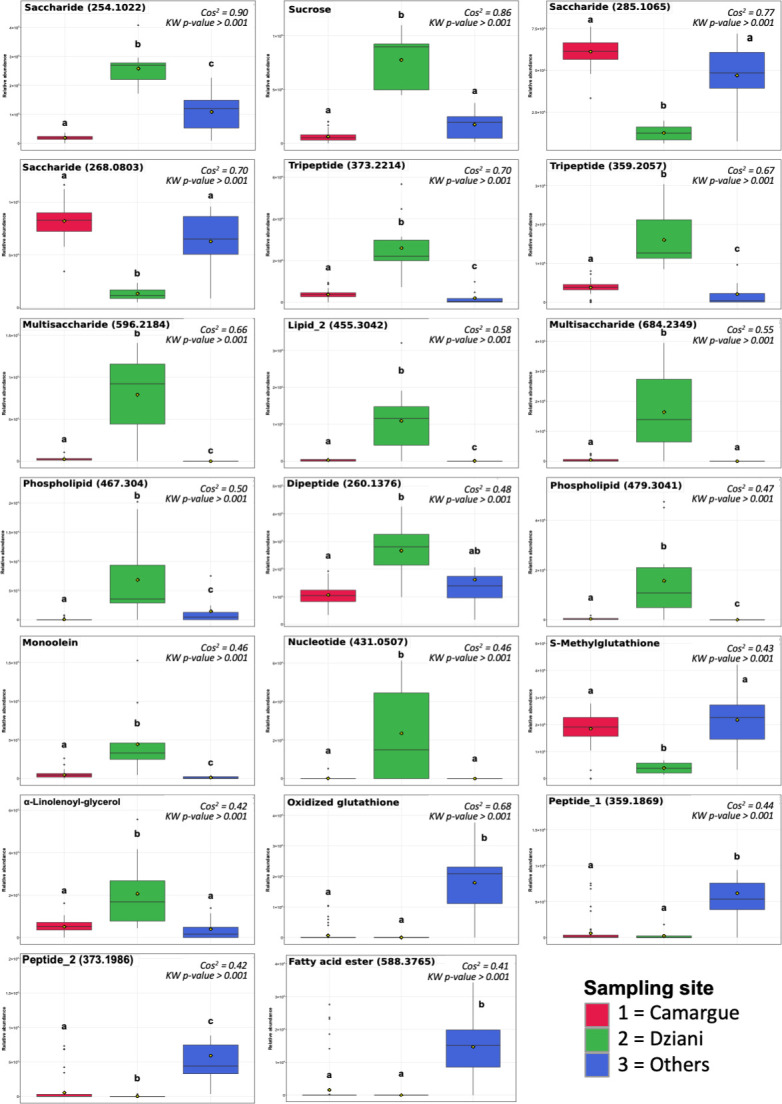
Relative abundances of most discriminated annotated ions (cos^2^ > 0.4) were compared between sampling sites (Kruskal-Wallis and pairwise Wilcoxon tests).

Six of the pigments were only present in the strains from Lake Dziani Dzaha (Fig. 1A and 4, Kruskal-Wallis test, *P* < 0.001). Among them, three were recognized as resembling caloxanthin, nostoxanthin, and an analog of β, β-carotene, and three were unknown carotenoids. The strains from Lake Dziani Dzaha did not possess the usual form of β, β-carotene, which was only present in strains from the Camargue and other locations. The allophycocyanins were significantly more abundant in the strains from other locations (Fig. 4, Kruskal-Wallis test, *P* < 0.001). Among the 20 most discriminated of the annotated metabolites (Fig. 5), 13 were significantly more abundant in the strains from Lake Dziani Dzaha, including 4 saccharides (MW = 254.1022, 596.2184, 684.2349 Da, and sucrose), 3 little peptides of wo or three amino acids (MW = 373.2214, 359.2057, and 260.1376 Da), 2 phospholipids (MW = 467.304, and 479.3041 Da), 1 lipid (MW = 455.3042 Da), and 2 unsaturated glycerol fatty acids annotated as monoolein and alpha-linolenoyl-glycerol (Kruskal-Wallis test, *P* < 0.001). Three molecules were significantly less abundant in the strains from Lake Dziani Dzaha: two saccharides (MW = 285.1065 and 268.0803 Da) and S-methylglutathione (Kruskal-Wallis test, *P* < 0.001). Four molecules were significantly more abundant in strains from other locations: two peptides (MW = 359.1869 and 373.1986 Da), one fatty acid ester (MW = 588.3765 Da), and oxidized glutathione (Kruskal-Wallis test, *P* < 0.001).

### Gene content

Among the 93 *Limnospira* strains investigated, 33 of them had available genomic information (Table S1) that could be explored for distinctive genetic traits related to the clustering of strains according to their chemical signatures. The results of a search for the presence of coding genes perfectly matching the distance patterns between the strains according to the core-genome sequence comparison allowed the distinguishing of two main groups of strains (I and II) (Fig. S3). The first group was divided into two subgroups (I.1 and I.2). Clade I.1 contained 23 strains from the Camargue region, and clade I.2 had four strains from Lake Dziani Dzaha, two strains from Lake Natron (*L. platensis* PMC 1042.18 and SAG 85.79), and the strain *L. platensis* PCC 8005. Clade II contained the last three strains of *L. platensis* PCC 7345, PMC 289.06, and Paracas R14.

These three coding gene clades (I.1, I.2, and II) can also be clearly discriminated according to both MFA and BC-MFA analyses (Fig. S4 A and B). The chemical composition of these three clades was mostly separated by the first axis of MFAs and BC-MFAs, while clade II was separated from clades I.1 and I.2 by the second axis of BC-MFAs. Among fatty acids, pigments, and annotated ions, 18 most discriminated molecules were observed on a BC-MFA variable plot to present a cos^2^ > 0.4 (Fig. S4C): 7 fatty acids, 7 pigments, and 4 annotated ions (17 related to axis 1 and 1 to axis 2). In total, 63 molecules showed significant differences between at least two groups (Kruskal-Wallis test, *P* < 0.01). The seven fatty acids, as well as the seven pigments, were less abundant in clade I.1 (Fig. S5, Kruskal-Wallis test, *P* < 0.01). Among the four most discriminated annotated ions, two saccharides (MW = 536.1602 and 430.133 Da) were significantly more abundant in the strains of clade I.1. The two other annotated ions were significantly more abundant in the strains of clade II: oxidized glutathione and a fatty acid ester (MW = 588.3765 Da) (Fig. S5, Kruskal-Wallis test, *P* < 0.01).

## DISCUSSION

In the present work, we have developed an original approach based on an investigation of the specific adaptations of the different chemotypes to the environmental conditions of their ecosystem of origin, through comparison with their regular chemical traits. Thus, we hypothesized that the specific chemical traits of these different genotypes might offer them strong adaptive features very likely based on genomic originalities shaped by the conditions of their originating environments.

Analyses of the contents of the pigments, lipids, and small metabolites of the various *Limnospira platensis* strains cultivated under identical laboratory conditions provided unprecedented data for obtaining an answer to the key question: is the diversity of these molecules driven by selective pressures on *Limnospira platensis* (clade I or II) (20) by the environment from which the strains were isolated? Our results indicate that the sampling location with its diverse environmental conditions of salinity, pH, light, and temperature constitutes the key variable explaining the distribution of *Limnospira platensis* strains into three distinct chemotype groups. The molecules that are most discriminatory for these three groups include saccharides, fatty acids, peptides, photosynthetic pigments, and antioxidant molecules. We hypothesize that these molecular enrichments reflect traits of physiological adaptations to conditions encountered in the sampling environments, potentially in relation to salinity, light, and oxidative stress.

### Osmoregulation and salinity resistance

The ponds of the Camargue in France and Lake Dziani Dzaha in Mayotte constitute environments with very different salinities. In the brackish ponds of the Camargue, close to the Mediterranean Sea, salinity varies between 12‰ and 35‰, while Lake Dziani Dzaha is a thalassohaline lake. Here, salinity is much higher, up to 70‰ (34). Strains from Lake Dziani Dzaha have greater sugar diversity and more long-chain polyunsaturated fatty acids compared with strains isolated from other habitats. They are also the only ones to produce nostoxanthin and caloxanthin. The specific chemical signature of strains from Lake Dziani Dzaha testifies to the adaptation of the *Limnospira platensis* species to resist the higher salinity in this specific environment.

In cyanobacteria in general, several families of molecules are involved in osmoregulation and detoxification of stress due to high salinity (35, 36). Cyanobacteria produce various sugars including glucose, sucrose, trehalose, and glycine betaine to prevent osmotic stress from high salinity (37). In *Synechococcus*, long-chain polyunsaturated fatty acids appear to be produced to a greater extent with increasing salinity to help combat the oxidative stress caused by high salinity (38). It has also been shown that certain carotenoids, nostoxanthin and caloxanthin, are produced by a bacterium of the genus *Sphingomonas* resistant to high salinity (39). In addition, the nostoxanthin- and caloxanthin-producing bacterium *Sphingomonas nostxanthinifaciens* may have a role in reducing the amount of reactive oxygen species (ROS) in *Arabidopsis thaliana* when the two organisms are co-cultured in high salt concentrations (40). Taken together, these findings support the idea that sugar and carotenoid molecules could represent an essential part of the high salinity adaptation traits of some *Limnospira platensis* strains.

### Light irradiance variations

In response to variations of light intensity and quantity, cyanobacteria can modify chlorophyll, phycobiliprotein, and carotenoid composition to efficiently optimize electron transfer and manage excess of energy (41–43). In *Synechococcus*, various carotenoids appear crucial for high light survival, as strains lacking carotenoid biosynthesis genes (*cru* genes) exhibit lower growth rates and higher ROS level contents compared with carotenoid-producing strains (41).

The different *Limnospira platensis* strains exhibit a large repertoire of diverse chlorophylls (*n* = 5–12) and carotenoids (*n* = 13–18) that could potentially be involved in peculiar photo-pigment adaptation. We notice that 18 of these carotenoid pigments present still unknown chemical structures, illustrating the remarkable the ability of *Limnospira* to exhibit peculiar and diver pigment contents, as previously shown by the characterization of up to 48 hydrophobic pigments (22 carotenoids and 26 chlorophylls) by liquid chromatography equipped with atmospheric pressure chemical ionization source coupled to Fourier Transform Ion Cyclotron Resonance Mass Spectrometry (LC-APCI-FT-ICR-MS) (44).

Thus, one of the most other noticeable results is the exclusive presence of six carotenoids in strains collected from Lake Dziani Dzaha. Remarkably, this lake of the south tropical area has an important mean surface irradiance of above 2,000 µmol photon∙s^−1^∙m^−2^, which remarkably decreases rapidly by 99% in the first 20 cm of surface water to around 20 µmol photon∙s^−1^∙m^−2^ (34). *Limnospira* living in the thin photic zone of this Lake are therefore subjected to both high light intensity at the upper surface and strong decrease in light over a relatively short depth when experiencing convection or migration movements through the photic water layer and even below. However, the potential involvement of the specific carotenoid content of genotype collected from lake Dziani for the resistance against light variation-induced stress remains theoretical and aims at being further tested by stress light experiments

### Oxidative stress

In general, high salinity, light intensity, and UV exposures are interdependently involved in ROS production and oxidative stress (45). In addition to the primary mechanisms listed above, cyanobacteria also produce a large variety of molecules other than carotenoids and long-chain polyunsaturated fatty acids aimed at reducing the adverse effects of oxidative stress. These molecules, including ergothioneine, glutathione, biopterins, and their derivatives, are specifically involved in reducing ROS production and combating the deleterious effects of oxidative stress (14, 46, 47). The cellular extracts of all *Limnospira platensis* strains currently investigated contained significant amounts of each of these molecules that might constitute a set of molecular resource for this organism to deal with a hazardous local cellular stressor.

### *Limnospira platensis* presents a peculiar ecological strategy

Cyanobacteria have colonized many types of habitats such as oceanic waters, fresh waters, and alkaline waters, either benthic or pelagic, and even terrestrial under symbiotic conditions (2). Aquatic species have developed various ecological strategies to adapt to their environment. Some cyanobacteria live in diversified communities of different species, responding to concomitant or consecutive proliferative events. These include benthic cyanobacteria, which form biofilms (e.g., *Phormidium*, *Lyngbya*, and *Oscillatoria*) (48), and pelagic cyanobacteria, which can form blooms (e.g., *Microcystis*, *Aphanizomenon*, and *Raphidiopsis*) (49–51). The genomes of these cyanobacteria are remarkably large (>5 Mb) and contain many biosynthetic gene clusters (BGCs) (52–54), producing various bioactive secondary metabolites including cyclic peptides, lipopeptides, alkaloids, macrolides, and polyketides that enable them to interact with their microbial community in terms of anti-grazing, anti-algal, communication blocking, and resource competition (5, 55). However, the production of these specific molecules has a high cellular energy cost because the synthetic pathways involve a large set of genes for the synthesis of many enzymes (56). In contrast, some marine picocyanobacteria such as *Prochlorococcus* and *Synechococcus* exclusively inhabit a single type of habitat, the worldwide oligotrophic seas, and exhibit a unique diversity of photosynthetic pigments, allowing them to exploit a wide range of micro-niches of varying light intensity (57, 58). The genomes of these cyanobacteria are remarkably small (1.5–2.5 Mb) and contain no BGCs (52, 59). In these cyanobacteria, adaptation to an ecological niche is encoded in both core and flexible parts of the genomes (60, 61).

For *Limnospira platensis*, we hypothesize a potential third strategy. *Limnospira platensis* genomes are relatively large, from 5.5 to 6.5 Mb (20, 62) being comparable to the genome size of community-dwelling cyanobacteria proliferating in eutrophic waters. Yet remarkably, they contain either no or only a single BGC of secondary metabolites (potentially involved in the biosynthesis of cyanobactins called Arthrospiramides), as do the specialized marine picocyanobacteria *Synechococcus* and *Prochlorococcus* (52, 63, 64). Unlike marine *Synechococcus* and *Prochlorococcus*, *Limnospira platensis* proliferates in many different aquatic habitats from fresh to brackish, alkaline, and saline alkaline waters (20, 65); *Limnospira platensis* strains belonging to the same phylogenetic clade do not share similar habitats (20). Moreover, in lakes where phytoplanktonic communities are dominated by *Limnospira platensis*, this species frequently generates persistent proliferations. These blooms are often accompanied by minimal surrounding diversity (except for the picoeukaryote, *Picocystis salinarum*) (21, 64), which is an indicator of extreme environmental conditions of hypersalinity, high light intensity coupled with high turbidity, elevated temperatures, high pH levels, and high sulfur contents. In such environments, only a limited number of microorganism clades can develop. This absence of surrounding microbial diversity, comprising potential competitors, could explain the absence of BGCs in the *Limnospira platensis* genomes, as it has fewer phototrophic organisms with which to interact through allelopathic compound production (52). Such an ecological strategy could support the high biomass production observed both *in situ* and *in vitro*, as lab culture conditions present many similarities (in terms of both biotic and abiotic factors) with those of the extreme environments in which *Limnospira* proliferates. Indeed, it has been shown by transcriptomics that the essential transcribed functions expressed *in vitro* are related to primary metabolism of sugars and fatty acids, levels of nitrogen and CO_2_, photosynthetic, and antioxidant processes (65).

Finally, we attempt to investigate the genomic distinction that could be retrieved from the comparison of the limited number of available genomes of strains originating from the different localities (20). Although genomes clearly show that both well-conserved (core) and flexible (pan) genome parts discriminate strains according to their respective origin locations (Fig. S3), their investigation does not allow to clearly identify genetic markers responsible of molecular discrimination observed at the pigment, fatty acid, and metabolite levels. Indeed, the genes specifically responsible of the synthesis of these molecules remain largely unknown, and filling these gaps of knowledge would still require further extensive experimental screening.

However, it can be presumed that the flexible part of the genome, which is remarkably rich in transposable and regulatory elements, contains a diverse set of genes, the molecular functions of most of which are still unknown and may dynamically support evolutionary adaptations such as ecological niche extension due to higher stress resistance (20). This may imply the synthesis of specific molecules, such as of small metabolite like redox agents, saccharides, and pigments/carotenoids, involved in ROS scavenging, osmoregulation, or photoprotection.

### Conclusions

Taken together, these ecological and evolutionary patterns provide a better understanding of the mechanisms that support the remarkable success of *Limnospira* in natural as well as lab or industrial environments. It is now essential to experimentally identify the genetic and phenotypic traits that have defined the different *Limnospira* ecotypes and to determine whether *Limnospira* clade I.1, I.2, and II genotypes can colonize similar ecological niches. This remarkable organism exhibits a ubiquitous geographic distribution that represents ecologically distinct and functionally distinguishable taxa. One key question remaining is to elucidate the mechanism that allows blooms of *Limnospira in situ* and *in vitro* to avoid density-dependent regulation of proliferation by cyanophages, as those encountered by other blooming cyanobacteria do (65,66). Such adaptive mechanisms would help explain how *Limnospira platensis* can form permanent blooms in many environments. To this end, two distinct hypotheses can be further tested: either the *Limnospira platensis* genome presents a mechanism to protect itself from phage lysis or the fraction of the population lysed by phages is replaced by rapidly dividing *Limnospira platensis*.

## Data Availability

All processed data are available in supplementary tables. Raw data are available upon request.

## SUPPLEMENTAL MATERIAL

Supplemental materials spectrum.01901-24-s0001.docx Supplemental methods and figures

Supplemental tables spectrum.01901-24-s0002.xlsx Tables S1 to S4.

ASM does not own the copyrights to Supplemental Material that may be linked to, or accessed through, an article. The authors have granted ASM a non-exclusive, world-wide license to publish the Supplemental Material files. Please contact the corresponding author directly for reuse.
