# Supplementary material for: Limnospira (Cyanobacteria) chemical fingerprint reveals local molecular adaptation
Source: Microbiol Spectr. 2025 Jan 8;13(2):e01901-24. doi: 10.1128/spectrum.01901-24 (PMC11792457; doi:10.1128/spectrum.01901-24)
Supplement: Supplemental materials — Supplemental methods and figures [file spectrum.01901-24-s0001.docx]

**Supplementary material and methods**

*Lipophilic pigment analysis*

Five mg of freeze-dried biomass were incubated with 95% methanol (buffered with 2% ammonium acetate) during 15 min, at -20 °C in the dark. Extracts were then filtered with 0.2 μm PTFE syringe filters and analyzed within 16 h using an Agilent 1260 Infinity HPLC composed of a quaternary pump (VL 400 bar), a UV–VIS photodiode array detector (DAD 1260 VL, 190–950 nm), a fluorescence detector (FLD 1260 excitation: 425 nm, emission: 655 nm), and a 100 μL automatic sample injector refrigerated at 4 °C in the dark. Chromatographic separation was carried out using a C_18_ column for reverse phase chromatography (Supelcosil, 25-cm long, 4.6 mm inner diameter). The solvents used were: 0.5 M ammonium acetate in methanol and water (85:15 v:v), acetonitrile and water (90:10 v:v), and 100% ethyl acetate. The solvent gradient was set according to Brotas and Plante-Cuny^28^, with a 0.5 mL.min^−1^ flow rate. Identification and calibration of the HPLC peaks were performed with β,β-carotene, canthaxanthin, chlorophyll *a*, diatoxanthin, diadinoxanthin, echinenone, and pheophytin *a* standards. We characterized and attempted to identify all detected peaks by their absorption spectra and relative retention times using the Agilent OpenLab software. Quantification was performed using standard calibration curves built with repeated injections of standards over a range of dilutions. Xanthophylls, carotenes and chlorophylls (other than *a*) were quantified at 470 nm, chlorophyll *a* and their derivatives as well as pheo-pigments were quantified at 665 nm. The relative abundance of each pigment (%) was calculated from its respective concentration in the sample (mg.g^-1^ dw for chlorophyll *a* derivative, and μg.g^−1^ dw for other pigments).

*Fatty acid analysis*

Complete procedure is detailed in supplementary material and methods. Samples were extracted with 20-min bath sonication a first time in a mixture of chloroform, water and methanol (1:1:2 v:v:v). One mL chloroform and one other of water were added, and the samples were then vortexed and centrifuged 5 min at 1,200 g. The lipids were retained in the chloroform phase, which was collected. Two mL chloroform was added to the samples before a second extraction with a sonicator bath (20 min). The samples were centrifuged again, then the chloroform was collected, and the total of 4 mL collected was evaporated under nitrogen (N_2_) flow. Lipids were saponified by adding a methanol:sodium hydroxide (2 N) mixture (2:1 v:v). Samples were placed at 90°C for 1h30 then 500 μL of HCl acid (37%) and 1.5 mL of CHCl_3_ were added. The tubes were then vortexed and centrifuged (1,200 g). The chloroform phase was collected and evaporated under N_2_ flow. Samples were methylated to form fatty acid methyl ester (FAME) by adding 1 mL boron trifluoride and then placed in a dry bath for 10 min at 90°C. One mL of H_2_O and 1 mL of chloroform were added. They were vortexed and centrifuged and then aqueous phase was pipetted and discarded. one mL of H_2_O and 1 mL of chloroform were added again, and the tubes were vortexed and centrifuged for 5 min (1,200 g).

All the chloroform containing Fatty acid methyl esters (FAME) was collected and placed in 2 mL vials. The vials were stored in a freezer (-20°C). FAME were quantified by gas chromatography (Agilent 8890 GC System) equipped with an Agilent VF-WAXms capillary column (30 m length × 0.25 mm inner diameter × 0.25 μm film thickness) and quantified using a flame ionisation detector (FID). The oven temperature is maintained at 60°C for 1 min, then it increases to 150°C (40°C.min^-1^) for 3 min and then to 240°C (3°C.min^-1^) for 19 min.

Fatty acids were identified with a mass spectrometer (Agilent 5977B GC/MSD) using the NIST mass spectral library and according to the comparison of retention times of commercial fatty acid standards (Supelco 37). The internal standard (23:0) was used to determine the concentration of each FA in µg per mg of freeze-dried gonad material. We then reported the values as % of total FA and concentration (mg.g^-1^ dw).

*Mass spectrometry metabolite analysis*

Five mg of freeze-dried biomass were extracted with 500 µL of methanol:water (75:25) with 0.1% of formic acid solution. Cell lysis was performed by sonication: 3 cycles of 30 s, 10 s break between each cycle, at 80% of the maximum intensity (SONICS Vibra Cell, Newton, CT, USA; 130 Watts, 20 kHz). Samples were then centrifuged (10 min, 13,400 g, 4°C), and the supernatants were collected and stored in obscurity at -20°C before mass spectrometry analysis. Extracts were separated using ultra-high performance liquid chromatography (UHPLC). For each sample, 2 μL was injected, and molecule separation was performed by a Polar Advance II 2.5 pore C_18_ (Thermo Fisher Scientific, Waltham, MA, USA) chromatographic column at a flow rate of 300 μL.min^−1^ under a linear gradient of acetonitrile acidified with 0.1% formic acid (from 5 to 90% in 15 min). Metabolites contents were analyzed using an electrospray ionization hybrid quadrupole time-of-flight (ESI-QqTOF) high-resolution mass spectrometer (Compact, Bruker, Bremen, Germany) in the range 50–1500 *m/z*. Compounds were initially analyzed in simple MS positive modes without quadrupole fragmentation. MS data were processed using MetaboScape 4.0 software (Bruker, Bremen, Germany) for recalibration of each sample analysis (according to internal standard), peak detection and selection of ions whose intensity was greater than 5,000 counts in at least 10% of the set of samples, and peak realignment. Furthermore, different states of charge and adducts were grouped together and the “area-under-the-peak” was determined in order to generate a unique global data matrix containing semi-quantification results for each metabolite in all analyzed samples’ peak for each analyte (characterized by the respective mean mass of its neutral form and its corresponding retention time). For qualitative investigation of metabolites, additional ion selection with the quadrupole and fragmentation was carried out in tandem by collision ion dissociation (CID) according to the autoMS/MS analysis, performed in positive mode. The top-intensity ions (> 5,000 counts in single MS (MS1)) were then selected by the quadrupole and fragmented in a collision cell (MS2). The resulting ions of the fragmentation of their respective parent were transferred and detected. The files containing the fragmentation information for each ion analyzed were exported in mgf format using MetaboScape 4.0 (Bruker, Bremen, Germany) software before being used for the generation of the molecular network of structural similarity. Metabolite annotations were made from MS2 data by generating a molecular network for the comparison of fragmentation profiles using the MetGem software (version 1.3.6) and the GNPS algorithm (Supplementary figure S1). Public and generalist spectral databases GNPS library, NIH Clinical collections, and EMBL metabolomics were used. Annotations were supplemented by a match with the CyanoMetDB database V1.0, which contains all the raw formulae of the above 2,100 cyanobacterial metabolites already described (Jones et al., 2021).

**Supplementary Figure S1-S5**

**Supplementary Figure S1.** T-*SNE* molecular network generated with Metgem 1.3.6 based on LC-MSMS dataset. The various colors show the different molecular families that constitute the different annotated clusters according to molecular fragment similarities with standards available in public databases (GNPS, HMDB, NIH and EMBL).

**Supplementary Figure S2.** PCA of the *Limnospira* strains analyzed for their pigment, lipid and metabolite contents according to the 3 sampling site groups and correspondant ANOSIM values.

**Supplementary Figure S3.** Dendrograms based of the 33 *Limnospira* available genomes according to genetic distance based on the sequence of 2672 most conserved genes of the core-genomes (left) and the presence/absence of coding genes (right). Distance is estimated using Euclidian method, depicted linkages is calculated as Ward method previously described in Roussel et al. (2023).

**Supplementary Figure S4.** Multiple factor analysis (MFA: A), and between class analysis with a cos^2^ threshold of 0.4 (BCA: B, cos^2^ plot: C) based on chemical composition of the strains. The tested factor is the coding gene clade.

**Supplementary Figure S5.** Most discriminant fatty acids, pigments and annotated ions (cos^2^ > 0.4) comparison between gene content clades (Kruskal-Wallis test, pairwise Wilcoxon testes). Pigment concentrations are given in mg.g^-1^ dw for phycobiliproteins and chlorophyll a, and in µg.g^-1^ dw for other pigments. Fatty acid concentrations are given in mg.g^-1^ dw. Untargeted ions are expressed in relative abundances.
